# Generational Differences in the Acceptance of Care Robots Among Portuguese Adults: Evidence from the Almere Model, ADL and IADL Frameworks

**DOI:** 10.3390/healthcare14162592

**Published:** 2026-08-18

**Authors:** Paula Tavares de Carvalho, Ricardo Jorge Raimundo, Nuno Piçarra

**Affiliations:** 1 BRU (IUL)—Business Research Unit, ISCTE—Instituto Superior de Ciências do Trabalho e da Empresa, 1649-026 Lisbon, Portugal; paula.t.carvalho@universidadeeuropeia.pt; 2 IADE—Faculdade de Design, Tecnologia e Comunicação, Universidade Europeia, 1200-649 Lisbon, Portugal; 3 ISCAL—Lisbon Accounting and Business School, IPL—Instituto Politécnico de Lisboa, 1069-035 Lisbon, Portugal; 4 ISEC Lisboa—Instituto Superior de Educação e Ciências, 1750-142 Lisbon, Portugal; 5 Faculdade de Ciências Humanas, Universidade Católica Portuguesa, 1649-023 Lisboa, Portugal

**Keywords:** care robots, socially assistive robots, human–robot interaction, technology acceptance, Almere Model, activities of daily living, instrumental activities of daily living, ageing, Industry 5.0, Society 5.0, older adults

## Abstract

Background: Population ageing, increasing care demands, and rapid advances in artificial intelligence and robotics have intensified interest in care robots as potential tools to support independent living and complement human caregiving. However, the successful implementation of robotic technologies depends largely on public acceptance, which is influenced by functional, psychological, ethical, cultural, and generational factors. Objective: This study examined generational differences in the acceptance of care robots among Portuguese adults by integrating the Almere Model of technology acceptance with the Katz Index of Activities of Daily Living (ADL) and the Lawton–Brody Instrumental Activities of Daily Living (IADL) Scale. The research sought to determine whether acceptance varies according to generation and the type of caregiving activity performed by the robot. Methods: A cross-sectional quantitative study was conducted using an online questionnaire administered to a purposive sample of 235 adults residing primarily in the Lisbon Metropolitan Area, Portugal. The questionnaire combined constructs from the Almere Model with perceptions of robotic assistance for ADLs and IADLs. Principal Component Analysis, reliability analysis, descriptive statistics, and inferential analyses were performed to examine differences across generational groups. Results: Acceptance of care robots was strongly task-dependent. Participants expressed significantly greater acceptance of robots assisting with instrumental activities, including housekeeping, shopping, transportation, meal preparation, and medication management, than with intimate personal care activities such as bathing, dressing, toileting, feeding, and continence care. Contrary to common assumptions regarding digital natives, Generation Z reported higher levels of fear, discomfort, and perceived intimidation than Generation X and Baby Boomers. Older generations generally demonstrated more pragmatic acceptance of robotic assistance, particularly regarding future support needs associated with ageing. Across generations, respondents preferred robots with more human-like appearances; however, emotional trust remained substantially lower than perceived functional usefulness. Conclusions: The findings suggest that acceptance of care robots is conditional rather than universal and is shaped by the nature of the caregiving task, generational differences, and broader emotional and cultural perceptions of care. Integrating the Almere Model with established ADL and IADL frameworks provides a novel perspective by linking technology acceptance to specific functional domains of caregiving. The results support the view that care robots are more likely to be accepted as complementary tools that enhance human-centred care rather than as substitutes for professional or family caregivers. Given the purposive and geographically limited sample, the findings should be interpreted cautiously and not generalised to the wider Portuguese population. They nevertheless provide valuable implications for the design of socially assistive robots, healthcare practice, and public policy in ageing societies.

## 1. Introduction

By 2050, the working-age population of Europe is projected to decline to approximately 364 million, representing a 25% reduction compared with 1995 levels, while the population aged 65 years and over is expected to increase from 101 million to nearly 173 million [1]. These demographic changes will place considerable pressure on healthcare systems, long-term care services, and informal caregiving networks, increasing the need for innovative solutions capable of supporting healthy ageing and independent living.

Portugal is among the European countries most affected by demographic ageing. Together with Italy, it is projected to remain one of the oldest populations in the European Union, characterized by a high proportion of older adults and a continuously increasing ageing index. Over the last decade, Portugal has experienced one of the largest increases in median age within the European Union, alongside Slovakia, Greece, and Italy, reflecting the rapid pace of demographic transformation [2]. This trend is expected to intensify demand for healthcare services while exacerbating existing shortages of professional and informal caregivers.

Within this context, advances in artificial intelligence (AI) and robotics have stimulated growing interest in care robots as complementary tools to support older adults. Consistent with the principles of Industry 5.0, care robots are increasingly viewed not as replacements for human caregivers but as technologies designed to augment human capabilities, promote independent living, facilitate health monitoring, improve communication, and reduce caregiver burden. Their role is therefore centered on enhancing person-centered care while preserving human dignity, autonomy, and well-being.

Despite these potential benefits, public acceptance of care robots remains uncertain. European citizens generally recognize the advantages of robotic technologies, particularly in professional environments, yet express greater reservations regarding their use in socially sensitive contexts, such as childcare and elder care [3]. These findings suggest that acceptance depends not only on technological capabilities but also on perceptions of trust, emotional appropriateness, and the nature of the care tasks performed.

Although previous studies have examined technology acceptance in healthcare, relatively little is known about how different generations perceive the use of care robots for supporting older adults, particularly within the Portuguese context. Moreover, existing research has rarely integrated technology acceptance with functional care needs, limiting understanding of whether acceptance varies according to the type of assistance provided.

To address this gap, the present study investigates generational differences in the acceptance of care robots among Portuguese adults. The study integrates three complementary frameworks. First, the Almere Model, developed by Heerink et al. [4], provides a validated framework for assessing the acceptance of socially assistive robots based on constructs derived from the Unified Theory of Acceptance and Use of Technology (UTAUT), including perceived usefulness, trust, anxiety, social influence, and behavioral intention. Second, the Katz Index of Activities of Daily Living (ADL) evaluates acceptance of robotic assistance for six fundamental self-care activities, namely bathing, dressing, toileting, transferring, continence, and feeding [5,6]. Third, the Lawton and Brody Instrumental Activities of Daily Living (IADL) Scale assesses acceptance of robotic support for more complex activities required for independent community living, including telephone use, shopping, meal preparation, housekeeping, laundry, transportation, medication management, and financial management [7,8].

By integrating these three frameworks, the study examines whether perceptions of care robots differ across generations according to both technology acceptance and the functional nature of the caregiving tasks. In doing so, it contributes to a better understanding of the social acceptance of care robots and provides evidence that may inform the design of socially assistive robots, healthcare practice, and public policies aimed at supporting ageing societies.

## 2. Literature Review

### 2.1. Industry 5.0 and Society 5.0: Human-Centred Technological Transformation

The emergence of Industry 5.0 represents an evolution beyond the automation-driven paradigm of Industry 4.0. While Industry 4.0 focused primarily on technologies such as artificial intelligence (AI), the Internet of Things (IoT), cyber-physical systems, big data, and cloud computing, Industry 5.0 places human well-being at the centre of technological innovation. Rather than replacing human capabilities, intelligent technologies are increasingly designed to augment human performance, improve quality of life, and promote sustainable social development [1,2,3,4].

According to the European Commission, Industry 5.0 is founded on three complementary principles: human-centricity, sustainability, and resilience [5]. Closely aligned with this vision, Society 5.0, introduced by the Japanese government, promotes the integration of intelligent technologies into everyday life to address major societal challenges, including demographic ageing, healthcare accessibility, and social inclusion [6].

Population ageing is one of the principal drivers of these developments. The World Health Organization projects that the global population aged 60 years and over will exceed 2 billion by 2050, with the number of people aged 80 years and over increasing substantially [7,8]. This demographic transition is expected to increase the prevalence of chronic diseases, functional dependence, and long-term care needs while intensifying shortages of healthcare professionals and informal caregivers [9]. Consequently, care robots and other socially assistive technologies are increasingly viewed as complementary tools capable of supporting independent living, reducing caregiver burden, and strengthening human-centred healthcare systems.

Within this context, artificial intelligence (AI), robotics, and intelligent assistive technologies have emerged as strategic components of future healthcare systems. Consistent with the principles of Industry 5.0, these technologies are increasingly viewed as collaborative tools that complement rather than replace healthcare professionals, supporting clinicians, caregivers, family members, and older adults through personalized assistance, continuous monitoring, and decision support [9,10]. Recent advances in machine learning, computer vision, natural language processing, and generative AI have significantly expanded the capabilities of intelligent care systems, enabling more adaptive, personalized, and context-aware support both in healthcare settings and in older adults’ homes.

The integration of robotics into healthcare also reflects a broader shift towards person-centred care, preventive medicine, and healthy ageing. Care robots can assist with routine activities, medication management, rehabilitation, cognitive stimulation, and social interaction, thereby supporting independent living while enabling healthcare professionals to focus on more complex clinical and interpersonal tasks. Nevertheless, the increasing use of AI in healthcare raises important ethical challenges related to privacy, transparency, accountability, trust, and human oversight. Consequently, the Industry 5.0 framework advocates the development of Responsible AI, ensuring that technological innovation remains aligned with human dignity, safety, equity, and societal well-being [11,12].

Within this human-centred perspective, care robots represent one of the most tangible applications of Industry 5.0 and Society 5.0. By integrating AI, robotics, and healthcare, they are designed to support functional independence, cognitive assistance, social engagement, and communication, while complementing rather than replacing human caregivers. The following section reviews the evolution, functions, and applications of care robots in elder care.

### 2.2. Population Ageing and the Transformation of Care Needs

Population ageing represents one of the most significant demographic transformations of the twenty-first century and poses major challenges for healthcare systems, long-term care, and public policy. According to the World Health Organization (WHO), the global population aged 60 years and over is projected to increase from approximately one billion in 2020 to more than two billion by 2050, while the number of people aged 80 years and above will almost triple during the same period [8]. Similarly, the United Nations estimates that by 2050 one in every six people worldwide will be aged 65 years or older, increasing demand for healthcare services and long-term care [9].

Population ageing is associated with higher prevalence of chronic diseases, multimorbidity, frailty, cognitive decline, and functional limitations, leading healthcare systems to shift from episodic treatment towards integrated, person-centred models emphasising prevention, chronic disease management, and community-based care [13]. Maintaining functional independence has therefore become a central objective of healthy ageing.

Functional ability is commonly assessed using two complementary instruments: the Katz Index of Activities of Daily Living (ADL) [6] and the Lawton and Brody Instrumental Activities of Daily Living (IADL) Scale [7]. The Katz Index evaluates an individual’s ability to perform essential self-care activities, including bathing, dressing, toileting, transferring, continence, and feeding [14], whereas the Lawton and Brody Scale measures more complex activities required for independent community living, such as telephone use, shopping, meal preparation, housekeeping, transportation, medication management, and financial administration [7]. Because instrumental activities generally decline before basic activities, these frameworks provide valuable indicators of functional independence and are particularly relevant for evaluating how assistive technologies may support older adults at different stages of dependency.

Increasing functional dependence also places considerable pressure on both informal caregivers and healthcare professionals, who face growing workloads resulting from demographic ageing and workforce shortages. Consequently, healthcare systems are increasingly adopting digital health technologies, including artificial intelligence, smart homes, wearable sensors, telemedicine, and socially assistive robots, to support ageing in place and complement conventional models of care [9,10,15].

Within this technological ecosystem, care robots occupy a distinctive role by combining physical assistance, cognitive support, health monitoring, and social interaction. Rather than replacing human caregivers, they are designed to promote autonomy, reduce caregiver burden, and enhance quality of life while supporting person-centred care. However, their successful implementation depends not only on technological capabilities but also on users’ trust, acceptance, and willingness to integrate robotic assistance into everyday life. Understanding these factors is therefore essential for assessing the future role of care robots in ageing societies.

### 2.3. Care Robots and Socially Assistive Technologies in Elder Care

The rapid ageing of the global population, increasing demand for long-term care, and persistent shortages of healthcare professionals have stimulated growing interest in care robots as technologies capable of supporting older adults while complementing conventional models of care. Over the past two decades, care robots have evolved from relatively simple assistive devices into intelligent systems integrating artificial intelligence, multimodal sensing, natural language processing, computer vision, and adaptive learning. This evolution reflects the broader transition towards human-centred healthcare, consistent with the principles of Industry 5.0 and Society 5.0 [10,11].

Unlike industrial robots designed to automate repetitive tasks, care robots are specifically developed to support functional independence, healthy ageing, and psychological well-being while assisting both formal and informal caregivers. Their role is not to replace healthcare professionals but to complement human care by providing assistance with daily activities, health monitoring, rehabilitation, communication, and social interaction [16]. Consequently, care robots are increasingly recognised as enabling technologies that support person-centred healthcare and independent living in ageing societies.

#### 2.3.1. Classification of Care Robots

The literature generally distinguishes between two broad categories of care robots: service robots and socially assistive robots (SARs). Service robots provide functional and physical assistance by supporting activities such as mobility, object transportation, environmental monitoring, medication management, rehabilitation, fall detection, emergency response, and smart home integration. Their primary objective is to help older adults maintain functional independence as physical capabilities decline.

In contrast, socially assistive robots (SARs) achieve therapeutic objectives primarily through social interaction rather than physical intervention. Broadbent et al. [17] define SARs as intelligent systems that support users through communication, companionship, motivation, and emotional engagement, while Feil-Seifer and Matarić [18] describe them as assistive technologies designed to influence users’ cognitive, emotional, and behavioural states through interpersonal interaction.

This distinction is particularly important because successful ageing depends not only on maintaining physical independence but also on preserving emotional well-being, social participation, cognitive functioning, and psychological resilience. Consequently, contemporary care robots increasingly combine functional assistance with social and emotional support, reflecting a more holistic and person-centred approach to healthy ageing.

#### 2.3.2. Evolution of Socially Assistive Robots

Early generations of socially assistive robots (SARs) relied primarily on pre-programmed responses and offered relatively limited interaction capabilities. Contemporary systems increasingly integrate artificial intelligence, including speech recognition, natural language processing, facial expression and emotion recognition, gesture interpretation, contextual reasoning, reinforcement learning, and Large Language Models (LLMs), enabling more adaptive and personalised interactions [12].

These advances allow care robots to engage in more natural conversations, remember previous interactions, personalise reminders, encourage physical activity, support cognitive rehabilitation, and facilitate communication with healthcare professionals and family members. Consequently, care robots are evolving from programmable assistive devices into intelligent social companions capable of adapting to users’ changing needs and fostering long-term, human-centred relationships [12].

#### 2.3.3. International Development of Care Robotics

The implementation of care robots has expanded considerably worldwide, although adoption strategies differ across regions [15]. Japan remains the global leader, driven by rapid population ageing, workforce shortages, and sustained government investment in robotic technologies for nursing homes, hospitals, and home care. China has accelerated the integration of AI-enabled care robots through national healthcare initiatives, focusing on rehabilitation, mobility support, smart monitoring, and socially assistive technologies. South Korea has prioritised rehabilitation robotics integrated with wearable sensors and AI-based systems, particularly for older adults recovering from neurological and musculoskeletal conditions. In Europe, collaborative Horizon-funded projects such as Care-O-bot, Robot-Era, HOBBIT, and GiraffPlus have promoted person-centred solutions supporting independent living, telepresence, and healthy ageing.

Despite these regional differences, a common objective underpins international developments: the use of care robots to support older adults’ autonomy, enhance quality of life, and complement the work of healthcare professionals rather than replace human care [15].

#### 2.3.4. Applications of Care Robots

Current evidence identifies four principal application domains for care robots: support for functional independence, health monitoring, caregiver assistance, and psychosocial support [10,11]. Care robots increasingly assist older adults with both Activities of Daily Living (ADLs) and Instrumental Activities of Daily Living (IADLs) by supporting tasks such as medication management, meal preparation, shopping, housekeeping, mobility, object retrieval, and emergency detection. These functions contribute to maintaining independence and may help delay institutionalization.

Advances in artificial intelligence further enable continuous monitoring of physiological indicators, behavioral changes, medication adherence, sleep quality, and physical activity, facilitating early intervention and improving communication between older adults and multidisciplinary healthcare teams [16,17]. At the same time, care robots help reduce caregiver burden by automating routine monitoring, providing reminders, supporting rehabilitation, and enhancing communication, thereby allowing healthcare professionals to focus on complex clinical decision-making and interpersonal care [18].

Beyond physical assistance, socially assistive robots play an important role in promoting emotional well-being. Studies involving robots such as Hyodol, Pepper, PARO, and GRACE have reported positive effects on loneliness, depressive symptoms, social engagement, cognitive stimulation, treatment adherence, and overall quality of life. For example, Sima et al. [19] found that emotional support mediated improvements in life satisfaction among older adults interacting with socially assistive robots, while Jung et al. [20] reported significant reductions in loneliness and depression following prolonged interaction with the companion robot Hyodol. Collectively, these findings suggest that emotional engagement constitutes one of the principal mechanisms through which socially assistive robots contribute to healthy ageing.

#### 2.3.5. Limitations of Current Evidence

Despite encouraging advances, systematic reviews continue to highlight important limitations in the existing evidence base. Most studies involve relatively small convenience samples, evaluate short-term interventions, use heterogeneous outcome measures, and provide limited evidence from multicentre randomised controlled trials [21]. Furthermore, practical implementation remains constrained by challenges related to technical reliability, usability, maintenance costs, interoperability, organisational readiness, privacy, ethical governance, trust, and digital literacy.

An important distinction emerging from recent research is that technological capability does not necessarily translate into social acceptance. Although care robots can effectively support medication management, health monitoring, and communication with caregivers, older adults may still be reluctant to accept them in intimate care settings because of concerns regarding privacy, autonomy, emotional authenticity, and trust [22,23]. These findings indicate that the successful implementation of care robots depends not only on technical performance but also on users’ perceptions, expectations, and willingness to interact with robotic technologies.

Overall, the literature suggests that care robots are most effective when they complement rather than replace human caregivers, combining functional assistance with meaningful social interaction. Consequently, understanding the acceptance of care robots requires moving beyond engineering perspectives towards theories that explain trust, interpersonal interaction, and behavioural intention. This need has contributed to the rapid development of Human–Robot Interaction (HRI) as an interdisciplinary field examining how individuals perceive, trust, communicate with, and collaborate with intelligent robotic systems. The following section therefore reviews the principal HRI theories underpinning the acceptance of care robots in elder care.

### 2.4. Human–Robot Interaction in Elder Care

The growing integration of artificial intelligence and socially assistive robots into healthcare has established Human–Robot Interaction (HRI) as a key interdisciplinary field combining robotics, artificial intelligence, psychology, gerontology, human factors engineering, and healthcare. HRI examines how individuals perceive, trust, communicate with, and collaborate with robotic systems, recognizing that successful implementation depends not only on technical performance but also on the quality of the social, emotional, and cognitive relationships developed between users and robots [11,24].

Unlike conventional assistive technologies, socially assistive robots engage in continuous interaction through verbal and non-verbal communication, adaptive behaviors, and personalized responses. As a result, users often attribute human-like characteristics, such as intentions, emotions, and social roles, to these systems, transforming technology use into an evolving interpersonal relationship. This social dimension distinguishes care robots from other digital health technologies and explains why HRI has become central to understanding their long-term acceptance and adoption in elder care [11,24].

#### 2.4.1. Evolution of Human–Robot Interaction

Early Human–Robot Interaction (HRI) research focused primarily on engineering challenges such as navigation, manipulation, obstacle avoidance, and operational safety. As robotic technologies became increasingly integrated into healthcare and domestic environments, researchers recognized that technical performance alone could not explain successful implementation. Robots capable of performing tasks efficiently were not necessarily accepted by users, particularly in care settings where trust, emotional comfort, dignity, and interpersonal communication play a fundamental role [25].

Consequently, HRI has evolved into a human-centered interdisciplinary field that draws on psychology, sociology, gerontology, communication, and ethics to explain how cognitive, emotional, social, and cultural factors influence interactions with robotic systems. This shift reflects the broader transition from Industry 4.0 to Industry 5.0, where the value of technological innovation is increasingly assessed according to its contribution to human well-being rather than automation alone.

#### 2.4.2. Trust as the Foundation of Human–Robot Interaction

Trust is consistently identified as one of the strongest determinants of successful Human–Robot Interaction (HRI) because it influences whether individuals are willing to rely on robotic recommendations, disclose personal information, delegate everyday tasks, and sustain long-term interactions [25]. Unlike conventional digital technologies, care robots possess physical embodiment, behavioral autonomy, and social presence, meaning that trust extends beyond technical reliability to include perceptions of competence, predictability, transparency, emotional security, and ethical behaviors.

Empirical studies reinforce the central role of trust in technology acceptance. Giorgi et al. [25] found that older adults expressed greater trust and willingness to cooperate with robots perceived as intelligent, while also valuing a degree of imperfection that enhanced perceived authenticity. Similarly, Ham and Maeng [26] demonstrated that trust mediates the relationship between perceived usefulness and behavioral intention, indicating that technological capabilities influence acceptance primarily through users’ psychological confidence. Collectively, these findings position trust as a key mechanism linking technological performance with the acceptance of care robots.

#### 2.4.3. Social Presence and Emotional Engagement

A second key construct within Human–Robot Interaction (HRI) is social presence, defined as the extent to which users perceive robots as meaningful social partners. This dimension is particularly relevant in elder care, where loneliness, bereavement, cognitive decline, depression, and social isolation can adversely affect quality of life.

Evidence suggests that socially assistive robots can foster emotionally supportive relationships. Jung et al. [20] reported that regular interaction with the companion robot Hyodol significantly reduced loneliness and depressive symptoms among community-dwelling older adults, while Sima et al. [19] found that emotional support was a key factor underlying improvements in life satisfaction. These findings indicate that emotional engagement is one of the principal mechanisms through which socially assistive robots contribute to healthy ageing.

Recent advances in Large Language Models (LLMs) further enhance this capability by enabling more natural, personalized, and context-aware interactions. Nevertheless, researchers emphasize that emotionally intelligent robots should complement rather than replace human relationships, avoiding excessive emotional dependence or unrealistic expectations regarding artificial empathy [11].

#### 2.4.4. Anthropomorphism and Robot Embodiment

Anthropomorphism represents another central concept within HRI. Users naturally attribute human characteristics, including personality, intentions, emotions, and intelligence, to socially interactive robots. Robot embodiment substantially shapes these perceptions. Ahmad et al. [16] compared three robotic embodiments (Nao, Miro and Vector) among older adults in Pakistan. Participants generally preferred the humanoid robot Nao, perceiving it as more trustworthy, familiar, and socially appropriate. However, acceptance depended not only on appearance but equally on language, cultural appropriateness, and perceived usefulness.

Comparable findings emerge from Jahn et al. [27], who compared the humanoid robot Ameca with a virtual avatar. While the humanoid robot generated stronger emotional engagement, some participants simultaneously experienced discomfort associated with highly human-like appearance.

These studies suggest that embodiment should be interpreted as a multidimensional construct encompassing physical appearance, behavioural expression, communication style, and perceived personality.

#### 2.4.5. Cultural Adaptation

Recent literature increasingly demonstrates that Human–Robot Interaction is profoundly influenced by culture. Communication styles, religious beliefs, family structures, language, interpersonal distance, and caregiving traditions all influence users’ expectations regarding robotic behaviour. Akinade et al. [28] argue that robots deployed within African healthcare settings should demonstrate cultural competence, adapting greetings, eye contact, gestures, and conversational styles according to local social norms. Likewise, Lima et al. [29] reported that conversational robots supporting dementia care in India required culturally adapted language, locally appropriate communication patterns, and greater sensitivity to family-centred decision-making. 

On the other hand, Ahmad et al. [16] reached similar conclusions in Pakistan, demonstrating that acceptance depended upon Urdu-language communication, religious sensitivity, and culturally familiar interaction. Altogether, these findings indicate that future socially assistive robots should become culturally adaptive rather than culturally neutral.

#### 2.4.6. Human–Robot Collaboration

As robotic capabilities expand, research increasingly focuses on Human–Robot Collaboration (HRC) rather than complete automation.

Healthcare professionals consistently prefer collaborative models in which robots perform repetitive monitoring, routine communication, logistics, and rehabilitation support while clinicians retain responsibility for diagnosis, empathy, ethical judgement, and complex decision-making. Ameur et al. [10] identify three complementary dimensions determining successful Human–Robot Collaboration: patient-centred interaction, healthcare professional collaboration and intelligent system performance. Likewise, Attard and Sultana [30] reported that healthcare professionals strongly preferred semi-autonomous collaborative robots over fully autonomous systems. These findings closely align with Industry 5.0’s emphasis on technology augmenting rather than replacing human expertise.

#### 2.4.7. Fear and Barriers to Interaction

Although acceptance of care robots generally increases following direct interaction, fear remains one of the principal barriers to adoption. Elsheikh et al. [23] identified recurring concerns among older adults, including privacy, reliability, technological complexity, emotional discomfort, dependence and unfamiliarity. Importantly, these concerns tend to diminish through repeated interaction, transparent communication, and participatory design. Similar patterns have been observed among healthcare professionals, whose concerns about role displacement decrease when robots are presented as collaborative assistants rather than replacements.

Overall, the Human–Robot Interaction (HRI) literature demonstrates that successful implementation of care robots depends as much on psychological, social, and cultural factors as on technological performance. Trust, social presence, emotional engagement, anthropomorphism, and transparency consistently emerge as key determinants of long-term acceptance. However, while HRI explains how users experience robotic systems, it does not fully explain why they ultimately decide to adopt or reject them. This requires technology acceptance models that examine behavioral intention through constructs such as perceived usefulness, ease of use, social influence, facilitating conditions, anxiety, and trust. Accordingly, the following section reviews the principal technology acceptance models, with particular emphasis on the Technology Acceptance Model (TAM).

### 2.5. Technology Acceptance of Care Robots

Successful implementation of care robots depends not only on technological sophistication but also on users’ willingness to adopt, trust, and integrate robotic systems into their daily lives. Thus, technology acceptance has become one of the most extensively investigated research domains within healthcare robotics, providing the theoretical foundation for understanding why older adults, caregivers, healthcare professionals, and healthcare organizations decide to accept or reject robotic technologies. Even though early research focused primarily on information systems, the emergence of socially assistive robots has substantially broadened the scope of technology acceptance by incorporating psychological, emotional, social, ethical, and cultural determinants that extend beyond traditional usability considerations [31,32].

Unlike conventional information technologies, socially assistive robots combine physical embodiment, autonomous behavior, artificial intelligence, and social interaction, making their acceptance considerably more complex than that of computers or mobile applications. Users do not merely evaluate robotic systems according to efficiency or ease of use; they also assess trustworthiness, emotional comfort, perceived intelligence, social appropriateness, privacy, and compatibility with personal values. Consequently, contemporary technology acceptance research increasingly integrates theories originating from Human–Computer Interaction, Human–Robot Interaction, psychology, gerontology, and communication sciences.

#### 2.5.1. Technology Acceptance Model (TAM)

The Technology Acceptance Model (TAM) proposed by Davis [31] remains the most influential theoretical framework explaining users’ adoption of new technologies. TAM argues that behavioral intention to use a technology is primarily determined by two cognitive beliefs: (a) Perceived Usefulness (PU), defined as the degree to which individuals believe that using a technology will improve task performance; and (b) Perceived Ease of Use (PEOU), referring to the degree to which individuals believe that technology can be used with minimal effort. These beliefs subsequently influence users’ attitudes and behavioral intentions, ultimately determining actual technology use.

Although originally developed for workplace information systems, TAM has been widely applied within healthcare robotics because older adults consistently evaluate care robots according to their perceived contribution to independent living, health management, safety, and emotional well-being. Numerous studies demonstrate that perceived usefulness remains the strongest predictor of acceptance, particularly when robots support medication management, communication, rehabilitation, cognitive stimulation, and daily activities [33,34]. Yet, researchers increasingly recognize that TAM alone cannot adequately explain acceptance of socially interactive robots because it omits emotional, relational, and social dimensions central to Human–Robot Interaction.

#### 2.5.2. Unified Theory of Acceptance and Use of Technology (UTAUT)

To overcome limitations of earlier acceptance models, Venkatesh et al. [32] proposed the Unified Theory of Acceptance and Use of Technology (UTAUT), integrating eight previous behavioral theories into a single explanatory framework. UTAUT identifies four principal determinants of behavioral intention: performance, expectancy, effort, expectancy, social influence, and facilitating conditions. The model additionally proposes that age, gender, experience, and voluntariness moderate technology acceptance.

Within elder care, UTAUT has proved particularly useful because acceptance frequently depends not only upon individual perceptions but also upon recommendations from healthcare professionals, family members, peers, and organizational support. Older adults frequently rely upon trusted caregivers when evaluating unfamiliar technologies, making social influence considerably more important than in conventional workplace information systems.

However, although UTAUT explains behavioral intention effectively, it does not explicitly consider anthropomorphism, emotional attachment, trust, or social presence, constructs that have become increasingly important within socially assistive robotics.

#### 2.5.3. The Almere Model

Recognizing the unique characteristics of socially interactive technologies, Heerink et al. [4] developed the Almere Model, specifically designed to explain acceptance of assistive social agents among older adults. Building upon TAM and UTAUT, the Almere Model incorporates additional constructs particularly relevant to Human–Robot Interaction, including: Anxiety, Trust, Social Presence, Perceived Sociability, Enjoyment, Facilitating Conditions, Social Influence, Perceived Adaptability and Behavioral Intention. 

Unlike traditional technology acceptance models, the Almere Model explicitly recognizes that socially assistive robots are expected to establish interpersonal relationships with users rather than simply perform functional tasks. This distinction makes the Almere Model particularly appropriate for studies investigating care robots within elder care settings. Recent validation studies consistently demonstrate that trust, enjoyment, perceived usefulness, and social influence remain among the strongest predictors of behavioral intention, while anxiety negatively influences adoption [4,35].

#### 2.5.4. Beyond TAM and Almere: Contemporary Acceptance Models

The rapid evolution of artificial intelligence has stimulated development of additional acceptance frameworks extending beyond classical technology acceptance theories.

First, Liu et al. [33] extended the Artificial Intelligence Device Use Acceptance (AIDUA) framework by incorporating: technology optimism, innovativeness and familiarity with AI. Their structural equation modelling demonstrated that technology optimism and innovativeness positively influence behavioral intention through performance expectancy and positive emotional responses, whereas perceived risk substantially reduces acceptance. These findings indicate that AI-specific psychological characteristics increasingly influence technology acceptance beyond traditional usability constructs.

Second, the Technology Usage Inventory (TUI) provides a multidimensional assessment of technology acceptance incorporating: usefulness, usability, accessibility, skepticism, emotional reactions, and behavioral intention. Friese et al. [36], for example, reported strong acceptance of service robots among nurses, with intention to use being positively associated with usability and usefulness while negatively associated with skepticism.

Third, developed by Bartneck et al. [37], the Godspeed Questionnaire Series evaluates users’ perceptions of robots across five dimensions: Anthropomorphism, Animacy, Likeability, Perceived Intelligence and Perceived Safety. Although not a technology acceptance model itself, the instrument has become one of the most widely used measures within Human–Robot Interaction because these perceptions substantially influence behavioral intention.

#### 2.5.5. Trust as a Mediating Mechanism

One of the most important developments within recent literature concerns recognition that trust functions as a mediating variable connecting technological capability with behavioral intention. Broadbent et al. [17] argued that healthcare robots must inspire confidence not only through technical reliability but also through predictable, socially appropriate behavior. Likewise, Ham and Maeng [26] demonstrated that trust mediates the relationship between perceived usefulness and willingness to adopt domestic humanoid robots. These findings reinforce previous Human–Robot Interaction research suggesting that behavioral intention depends upon psychological confidence rather than technological performance alone.

#### 2.5.6. Acceptance Across Generations

An emerging body of research suggests that technology acceptance may vary across generations. Younger individuals generally report higher digital literacy and greater familiarity with artificial intelligence, whereas older adults tend to express stronger concerns regarding privacy, reliability, autonomy, and emotional authenticity. However, several studies indicate that these differences diminish following direct interaction with socially assistive robots, suggesting that experience can modify initial perceptions and increase acceptance over time [23,34]. This evidence provides a theoretical rationale for examining generational differences in the acceptance of care robots.

Overall, technology acceptance research has evolved beyond the original Technology Acceptance Model (TAM) by recognising that socially assistive robots introduce additional determinants, including trust, social presence, anthropomorphism, emotional engagement, and ethical transparency. Among the available frameworks, the Almere Model provides one of the most comprehensive approaches to explaining the acceptance of care robots because it integrates traditional technology acceptance constructs with Human–Robot Interaction variables specifically developed for socially interactive technologies. Accordingly, the present study adopts the Almere Model as its principal theoretical framework and combines it with the Katz Index and the Lawton–Brody Scale to examine whether perceptions of care robots differ across generations according to both technology acceptance and the type of caregiving activity.

### 2.6. Trust, Ethics and Responsible Artificial Intelligence in Care Robotics

The rapid integration of artificial intelligence into healthcare robotics has substantially increased the importance of trust, ethical governance, and Responsible Artificial Intelligence (RAI) as fundamental determinants of successful implementation. Although technological capabilities continue to improve through advances in machine learning, computer vision, natural language processing, and Large Language Models (LLMs), empirical evidence consistently demonstrates that technological sophistication alone does not ensure users’ willingness to adopt robotic systems. Rather, acceptance depends upon whether individuals perceive robots as trustworthy, transparent, safe, ethically responsible, and compatible with their personal values [10,11].

Unlike conventional healthcare technologies, socially assistive robots continuously interact with users while collecting sensitive behavioral, physiological, conversational, and environmental information. Consequently, older adults evaluate robotic systems not only according to functional performance but also according to broader ethical considerations including privacy, dignity, autonomy, emotional authenticity, accountability, and fairness. These dimensions have become increasingly important as robotic systems acquire greater autonomy through artificial intelligence and adaptive learning algorithms.

#### 2.6.1. Trust in Care Robotics

Trust has become one of the most extensively investigated constructs within Human–Robot Interaction and technology acceptance research. Mayer et al. [38] conceptualize trust as the willingness of an individual to become vulnerable to another party based on expectations regarding competence, integrity, and benevolence. Although originally developed within organizational psychology, this conceptualization has been widely adopted within Human–Robot Interaction because socially assistive robots increasingly function as autonomous partners capable of influencing users’ decisions and behaviors.

Within healthcare robotics, trust extends beyond technical reliability to encompass multiple complementary dimensions: competence, predictability, transparency, safety, emotional reliability and ethical behavior.

Broadbent et al. [17], in contrast, argued that healthcare robots must establish confidence through both technical performance and socially appropriate interaction. In the same vein, Dautenhahn [39] emphasized that long-term Human–Robot Interaction depends upon the development of predictable and socially meaningful relationships rather than simple task execution. Moreover, recent empirical evidence reinforces these conclusions. Giorgi et al. [25] demonstrated that perceived intelligence significantly increased trust among older adults interacting with multiple robots, while Ham and Maeng [26] showed, on the other hand, that trust mediated the relationship between perceived usefulness and behavioral intention towards domestic humanoid robots. Together, these studies indicate that trust constitutes the central psychological mechanism linking technological capability with long-term acceptance.

#### 2.6.2. Ethical Principles in Elder Care Robotics

As robots become increasingly autonomous, ethical considerations have become inseparable from technological development. Healthcare robotics literature consistently identifies several fundamental ethical principles [3]:

Respect for Autonomy: Older adults should retain control over decisions regarding robot use, data sharing, and levels of assistance, while robots should support autonomy rather than encourage unnecessary dependence.

Beneficence: Robotic interventions should demonstrably improve health, well-being, safety, and quality of life. Their deployment should generate measurable benefits for users.

Non-maleficence: Robotic systems should minimize physical risks, psychological harm, emotional manipulation and algorithmic errors. Lastly, safety validation therefore remains essential before widespread implementation.

Justice: access to robotic technologies should remain equitable regardless of age, socioeconomic status, education, or geographical location. Researchers increasingly emphasize that intelligent healthcare should reduce rather than reinforce health inequalities.

Respect for Human Dignity: perhaps the most distinctive ethical issue concerns preserving dignity during intimate caregiving activities. Although robots may successfully support hygiene, feeding, or mobility, many users remain reluctant to delegate emotionally sensitive activities entirely to autonomous systems. Thus, robots are increasingly conceptualized as assistants rather than replacements for human caregivers.

#### 2.6.3. Privacy and Data Governance

Modern care robots continuously collect multiple categories of sensitive information, including: speech, facial expressions, physiological measurements, behavioral routines, medication adherence, mobility patterns, and environmental data. These data facilitate personalized healthcare but simultaneously introduce concerns regarding: privacy, cybersecurity, informed consent, data ownership and algorithmic bias.

In addition, Osnat’s [13] global review reported that privacy concerns remain one of the strongest barriers to AI adoption despite widespread recognition of potential healthcare benefits. Likewise, Elsheikh et al. [23] found that concerns regarding surveillance and misuse of personal information contributed significantly to fear of social robots among older adults. As a result, transparent governance frameworks have become essential for maintaining public trust.

#### 2.6.4. Explainable Artificial Intelligence

The emergence of Large Language Models has substantially transformed socially assistive robotics [23]. Unlike earlier rule-based systems, contemporary robots increasingly generate adaptive responses based upon probabilistic reasoning rather than predefined algorithms. Although these capabilities improve conversational quality, they also reduce transparency. As a result, researchers increasingly advocate adoption of Explainable Artificial Intelligence (XAI).

Explainable AI aims to ensure that robotic systems can communicate why recommendations were made, how decisions were reached, and the levels of uncertainty and confidence associated with predictions. Within healthcare, explainability becomes particularly important because users may rely upon robotic recommendations when making decisions regarding medication, rehabilitation, nutrition, or emergency assistance. In short, explainability has become an essential component of trustworthy AI.

#### 2.6.5. Responsible Artificial Intelligence

The European Commission increasingly promotes Responsible AI as a central pillar of Industry 5.0 [3]. Responsible AI integrates technological innovation with: transparency, accountability, fairness, privacy, human oversight and sustainability. Rather than maximizing automation, Responsible AI seeks to ensure that intelligent systems remain aligned with human values throughout their lifecycle. This perspective closely corresponds with the World Health Organization’s recommendations concerning ethical AI for healthcare, emphasizing protection of human autonomy, promotion of well-being, transparency, responsibility, inclusiveness, and sustainability [8]. Within elder care, Responsible AI implies that robotic systems should continuously support, not replace, human judgement while preserving older adults’ dignity, independence, and social participation.

#### 2.6.6. Trust and the Almere Model

Trust occupies a central position within the Almere Model, interacting with constructs such as perceived usefulness, social presence, enjoyment, facilitating conditions, and behavioural intention [4]. Recent research consistently identifies trust as one of the strongest predictors of willingness to use care robots, user satisfaction, and long-term acceptance, whereas privacy concerns, anxiety, lack of transparency, and technological uncertainty reduce behavioural intention.

Given that the present study examines generational differences in trust towards care robots, the inclusion of trust as a key analytical dimension is supported by both the Human–Robot Interaction (HRI) literature and the Almere Model. As care robots become increasingly autonomous through artificial intelligence, acceptance depends not only on technical performance but also on whether these technologies are perceived as trustworthy, transparent, respectful of privacy, and supportive of users’ autonomy. This perspective is consistent with the principles of Industry 5.0, which emphasise technologies that complement rather than replace human care.

Building on this theoretical foundation, the following section examines the functional domains in which care robots may provide practical value, focusing on Activities of Daily Living (ADLs) and Instrumental Activities of Daily Living (IADLs), represented by the Katz Index and the Lawton and Brody Scale, respectively.

### 2.7. Functional Independence in Older Adults: Activities of Daily Living and Instrumental Activities of Daily Living

Maintaining functional independence is widely recognised as a fundamental determinant of healthy ageing, quality of life, and personal autonomy among older adults. According to the World Health Organization (WHO), healthy ageing involves developing and maintaining the functional ability that enables well-being in later life, reflecting the interaction between individuals’ physical and cognitive capacities and their surrounding environment [8]. Consequently, preserving functional independence has become a central objective of geriatric care, rehabilitation, and person-centred healthcare, particularly within the context of Industry 5.0, which promotes technologies that support autonomy rather than replace human capabilities.

In gerontology, functional independence is commonly assessed through two complementary domains: Activities of Daily Living (ADLs) and Instrumental Activities of Daily Living (IADLs). While closely related, these constructs represent different levels of functional complexity and dependency, requiring distinct forms of support. This distinction is particularly relevant when evaluating the potential contribution of care robots, as different robotic functions may assist older adults according to the specific activities they are no longer able to perform independently.

#### 2.7.1. Activities of Daily Living (ADLs): The Katz Index

The Katz Index of Activities of Daily Living (ADLs), developed by Katz et al. [5], is one of the most widely used instruments for assessing basic functional independence in older adults. It evaluates an individual’s ability to perform six essential self-care activities—bathing, dressing, toileting, transferring, continence, and feeding—which represent the minimum functional requirements for independent living. Declining ADL performance is closely associated with frailty, disability, institutionalisation, and increased healthcare utilisation.

In the context of care robotics, ADLs constitute one of the most challenging application domains because they involve intimate physical assistance and raise important ethical issues related to dignity, privacy, safety, and autonomy. Although care robots have demonstrated increasing capabilities in supporting mobility, rehabilitation, and fall prevention, users generally show greater acceptance when robots complement rather than replace human caregivers, assisting with physical support, reminders, and communication while maintaining human supervision during intimate care activities [17].

#### 2.7.2. Instrumental Activities of Daily Living (IADLs): The Lawton and Brody Scale

While ADLs evaluate fundamental self-care, the Lawton and Brody Instrumental Activities of Daily Living Scale assesses more complex activities required for independent community living [6]. The original scale evaluates an individual’s capacity to perform: telephone use, shopping, food preparation, housekeeping, laundry, transportation, medication management and financial management. Unlike ADLs, Instrumental Activities of Daily Living require higher levels of executive functioning, cognitive ability, planning, memory, and social participation. Consequently, IADLs often decline earlier than basic self-care activities and therefore provide sensitive indicators of early functional deterioration.

From the perspective of care robotics, IADLs constitute one of the domains in which intelligent technologies currently demonstrate the greatest practical potential. Examples include: medication reminders, navigation assistance, shopping support, financial reminders, appointment scheduling, smart home integration, communication with healthcare professionals and transportation assistance. Many of these activities depend primarily upon cognitive support rather than physical manipulation, making them particularly suitable for AI-enabled socially assistive robots capable of combining conversational interaction with intelligent decision support. Recent advances in artificial intelligence have further expanded these capabilities.

#### 2.7.3. Functional Independence and Care Robotics

The distinction between ADLs and IADLs has important implications for understanding acceptance of care robots. Evidence consistently suggests that users demonstrate greater acceptance of robotic assistance for Instrumental Activities of Daily Living than for Basic Activities of Daily Living [17].

Several explanations have been proposed. First, IADLs generally involve organisational or cognitive assistance rather than intimate physical care. Second, users perceive many IADL-related tasks as less threatening to personal dignity and autonomy. Third, intelligent technologies already demonstrate high levels of performance in scheduling, reminders, communication, monitoring, and information management. In reverse, ADLs frequently involve physical contact, personal hygiene, or intimate care, generating greater ethical sensitivity and stronger concerns regarding privacy, emotional comfort, and safety.

Broadbent et al. [17] argued that older adults generally express greater willingness to accept robotic assistance for household tasks than for intimate caregiving activities. Similar conclusions emerge from more recent Human–Robot Interaction studies, which consistently demonstrate that perceived usefulness varies according to the specific activity being supported rather than reflecting general attitudes towards robotics. These findings suggest that acceptance should not be conceptualised as a single global construct but rather as a domain-specific evaluation depending upon the nature of the activity being performed.

#### 2.7.4. Functional Independence, Successful Ageing and Industry 5.0

Maintaining functional independence extends beyond reducing healthcare costs or delaying institutionalisation. Contemporary theories of successful ageing emphasise autonomy, participation, dignity, social inclusion, and quality of life as central outcomes. Within this perspective, care robots should not be evaluated solely according to their technical capabilities but according to their contribution to preserving older adults’ functional ability and supporting independent living.

This perspective aligns closely with Industry 5.0 and Society 5.0, which advocate human-centred technological innovation designed to augment rather than replace human capabilities. Intelligent robotic systems therefore become enabling technologies that support older adults in maintaining autonomy while preserving meaningful relationships with caregivers, healthcare professionals, family members, and society.

#### 2.7.5. Functional Independence and the Present Study

The distinction between Activities of Daily Living (ADLs) and Instrumental Activities of Daily Living (IADLs) provides the conceptual foundation for the first two hypotheses of this study. While previous research indicates that older adults generally show greater acceptance of robotic assistance for IADLs than for ADLs, relatively few studies have examined whether these perceptions vary across generational cohorts. Because generations differ in digital literacy, technological familiarity, trust, and perceived usefulness, they may also differ in their willingness to delegate specific daily activities to care robots.

Accordingly, the present study distinguishes between perceptions of robotic assistance for Basic Activities of Daily Living, assessed using the Katz Index, and Instrumental Activities of Daily Living, assessed using the Lawton and Brody Scale. This distinction enables a more nuanced understanding of care robot acceptance by recognizing that willingness to use robotic assistance may depend on the functional complexity, intimacy, and ethical sensitivity of the caregiving task. These considerations provide the theoretical rationale for examining whether perceptions of robotic support for ADLs and IADLs differ across generations.

### 2.8. Research Gap and Rationale for the Portuguese Case

The literature demonstrates relevant growth in research on care robots, technology acceptance, and human–robot trust. Nevertheless, several gaps remain. First, much of the literature is concentrated in Asia, Northern Europe, or experimental lab contexts. Second, scarcer studies focus on Southern European countries, where demographic aging is high but cultural attitudes toward family care could differ. Third, most studies explore acceptance theoretically, without linking it to real levels of independence in daily living.

Portugal provides an appropriate case because it combines rapid demographic aging with a European cultural context where family care remains noteworthy. It is also a country where social acceptance of care robots cannot be assumed. Investigating attitudes in Portugal in 2026 therefore contributes both empirically and conceptually. It tests whether the human-centric promises of Industry 5.0 and Society 5.0 reverberate in a real social context and if robotic elder care is perceived as a value-adding assistance or an undesirable substitute for human care.

## 3. Materials and Methods

### Study Design, Questionnaire Development and Sample

This cross-sectional quantitative study was conducted in the Lisbon Metropolitan Area, Portugal’s largest urban region, with approximately 2.87 million inhabitants, representing around one-quarter of the national population. The region concentrates more than 40% of the country’s public and private sector employment, making it a socioeconomically diverse setting and an appropriate context for examining perceptions of care robots across different generations [2]. Although the study was geographically limited to the Lisbon Metropolitan Area, the objective was not to produce nationally representative estimates but rather to explore generational differences in attitudes towards the use of care robots for supporting older adults.

Data were collected using a structured self-administered questionnaire specifically designed to investigate perceptions of care robots in the context of elder care. Before accessing the questionnaire, participants were presented with an introductory section explaining the purpose of the study, the voluntary nature of participation, confidentiality and anonymity procedures, and the contact details of the research team. Informed consent was obtained electronically before participants proceeded to the survey.

The questionnaire comprised five sections. The first collected sociodemographic and occupational information, including age, gender, educational attainment, marital status, employment contract, years of professional experience, occupational sector, hierarchical position, and monthly income. The second explored participants’ health status, concerns regarding ageing, and attitudes towards health-related technologies. The third and fourth sections assessed perceptions of robotic assistance in relation to Instrumental Activities of Daily Living (IADLs) and Basic Activities of Daily Living (ADLs) using items derived from the Lawton–Brody Scale and the Katz Index, respectively. The fifth section measured participants’ acceptance of care robots using constructs adapted from the Almere Model, including perceived usefulness, trust, perceived ease of use, anxiety, social influence, and behavioral intention.

All attitudinal items were measured using a five-point Likert scale, ranging from 1 (strongly disagree) to 5 (strongly agree). For descriptive purposes, mean scores below 3.0 were interpreted as indicating disagreement, scores around 3.0 as neutral perceptions, and scores above 3.0 as agreement. These thresholds were used solely to facilitate interpretation and were not applied in the inferential statistical analyses.

A purposive sampling strategy was adopted to recruit participants from different generational cohorts while ensuring the inclusion of younger respondents expected to have substantial exposure to digital technologies and artificial intelligence applications. In particular, Generation Z participants were recruited primarily from university programs in Healthcare (30%), Marketing and Advertising (40%), and Hospitality Management (30%). These disciplines were selected because they represent sectors undergoing significant technological transformation through the adoption of artificial intelligence, automation, and service robotics. Healthcare and hospitality are additionally characterized by increasing workforce shortages and demographic pressures, making them particularly relevant contexts for investigating future acceptance of care robots. Marketing and advertising were included because of their rapid integration of AI-enabled technologies and their students’ familiarity with emerging digital innovations.

The purposive sampling approach was considered appropriate for this exploratory study because it enabled the inclusion of participants with differing levels of anticipated exposure to robotic technologies and future care environments. Nevertheless, the resulting sample included a relatively large proportion of Generation Z respondents, which should be considered when interpreting comparisons across generations. This limitation is discussed further in Section 7.

A total of 235 questionnaires were received. Following data screening for completeness and consistency, 217 valid responses were retained for analysis. The demographic characteristics of the final sample are presented in Table 1.

## 4. Results

The study aimed to examine whether generational differences exist in the acceptance of care robots among Portuguese adults. Specifically, it investigated whether perceptions varied across generations regarding the potential contribution of robots to supporting Instrumental Activities of Daily Living (IADLs), Basic Activities of Daily Living (ADLs), and broader technology acceptance, as conceptualized by the Lawton–Brody Scale, the Katz Index, and the Almere Model, respectively. Data were analyzed using IBM SPSS Statistics version 29 (IBM Corp., Armonk, NY, USA).

The Lawton and Brody Instrumental Activities of Daily Living (IADL) Scale is a widely used geriatric assessment instrument that evaluates an individual’s ability to perform complex daily activities necessary for independent living within the community. These activities include, among others, using the telephone, shopping, meal preparation, housekeeping, transportation, medication management, and financial management. In contrast, the Katz Index of Activities of Daily Living (ADL) assesses more fundamental self-care activities, including bathing, dressing, toileting, transferring, continence, and feeding. Together, these two instruments provide a comprehensive assessment of functional independence by distinguishing between basic and instrumental activities associated with everyday living.

To complement these functional assessment frameworks, the study employed the Almere Model, a technology acceptance framework specifically developed to evaluate older adults’ acceptance of socially assistive robots. The model extends traditional technology acceptance theories by incorporating constructs particularly relevant to Human–Robot Interaction, including perceived usefulness, trust, anxiety, perceived sociability, enjoyment, social influence, and behavioral intention. Integrating the Almere Model with the Katz and Lawton–Brody frameworks enabled the present study to examine technology acceptance in relation to specific caregiving activities rather than as an abstract attitude towards robotics.

**Figure 1:**
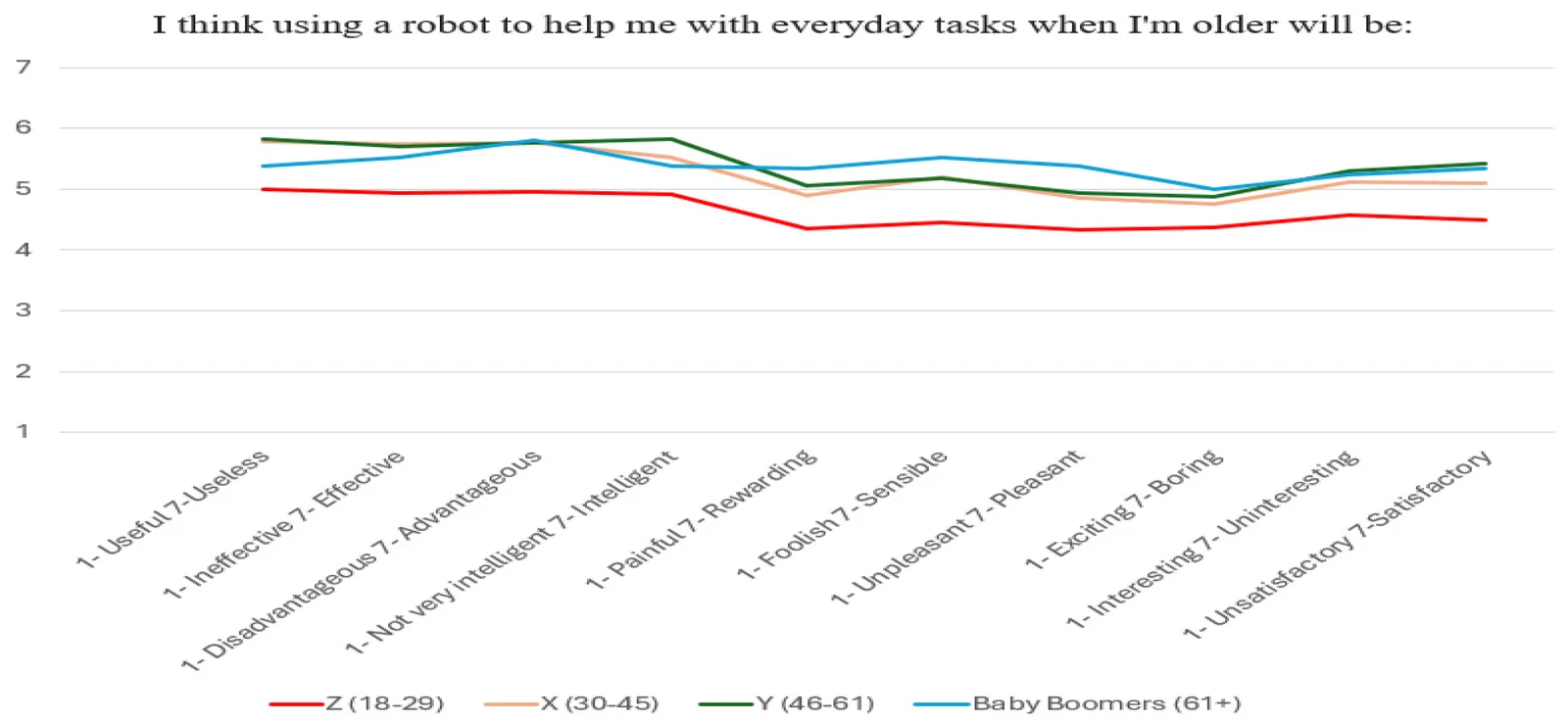
Almere Model.

Participants also evaluated the statement, “I think using a robot to help me with everyday tasks when I am older will be…”, using a seven-point semantic differential scale, where 1 represented the most negative evaluation and 7 the most positive. The corresponding results are presented in Figure 1. Overall, respondents across all generational cohorts recognized the potential usefulness of care robots in supporting everyday activities during older adulthood. Nevertheless, important intergenerational differences emerged. Although all groups reported generally positive perceptions, Generation Z consistently recorded the lowest mean scores, indicating a comparatively more cautious or reserved attitude towards the future use of care robots. These findings suggest that greater familiarity with digital technologies does not necessarily translate into higher acceptance of embodied robotic systems in caregiving contexts, a result that is explored further in Section 5.

The present study seeks to determine whether generational differences exist in attitudes towards the use of care robots for supporting older adults. Specifically, it investigates whether these differences are observed across three complementary dimensions: functional support for Instrumental Activities of Daily Living (IADLs), assessed using the Lawton and Brody Scale; functional support for Basic Activities of Daily Living (ADLs), assessed using the Katz Index; and technology acceptance, assessed using the Almere Model. Accordingly, the study addresses the following research question:

RQ: Do generational cohorts differ in their perceptions of the usefulness and acceptance of care robots for supporting older adults across the functional domains represented by the Lawton and Brody Scale, the Katz Index, and the Almere Model?

Based on the literature reviewed, the following hypotheses were formulated:

**Hypothesis** **H1.** *Generational cohort significantly influences perceptions of the usefulness of care robots for supporting Instrumental Activities of Daily Living (IADLs).*

**Hypothesis** **H2.** *Generational cohort significantly influences perceptions of the usefulness of care robots for supporting Basic Activities of Daily Living (ADLs).*

**Hypothesis** **H3.** *Generational cohort significantly influences the acceptance of care robots, including perceived usefulness and trust, as conceptualized by the Almere Model.*

To test these hypotheses, one-way analysis of variance (ANOVA) was performed to examine whether mean scores differed significantly across generational cohorts. Prior to conducting the ANOVA, the assumptions of normality and homogeneity of variances were evaluated. Homogeneity of variances was assessed using Levene’s test, with a non-significant result (*p* > 0.05) indicating that the assumption was satisfied. When significant differences between group means were identified, post hoc multiple comparison tests were performed to determine which generations differed significantly. Scheffé and Bonferroni tests were applied when the assumption of equal variances was met, whereas Dunnett’s C test was used when homogeneity of variances was violated. Statistical significance was established at *p* < 0.05.

In addition to statistical significance, effect sizes (η^2^) were calculated to assess the practical magnitude of the observed differences across generational cohorts. Where appropriate, 95% confidence intervals are also reported to facilitate interpretation of the results.

### 4.1. The Lawton and Brody Scale (Also Known as the Lawton Index)

Different Generations: Cross study.

To test H1, mean differences among generational cohorts were examined for variables related to the perceived usefulness of care robots in supporting Instrumental Activities of Daily Living (IADLs). A summary of the descriptive statistics and mean comparisons is presented in Table 2.

### 4.2. H1: Generational Differences in Perceptions of Care Robots Supporting Instrumental Activities of Daily Living (IADLs)

With regard to Instrumental Activities of Daily Living (IADLs), the analysis revealed no statistically significant differences between generational cohorts for most of the activities assessed. Across generations, respondents generally disagreed with the use of robots to make telephone calls on their behalf, manage their medication, provide companionship, or handle financial matters. Conversely, respondents generally agreed that robots could assist with shopping, meal preparation, and transportation.

However, statistically significant generational differences (*p* < 0.05) were identified for three activities:

- Helping to use the telephone: Generation X expressed significantly greater acceptance of robotic assistance for telephone use than Generation Z, which reported comparatively lower levels of agreement.
- Cleaning the house: Although all generations expressed positive attitudes towards robotic assistance with household cleaning, Generation X and Generation Y reported significantly higher levels of acceptance than Generation Z.
- Doing the laundry: Acceptance of robotic assistance with laundry also differed significantly across generations. While all groups were generally supportive of this application, Generation Y reported the highest level of acceptance, followed by Baby Boomers, whereas Generation Z expressed comparatively lower levels of agreement.
- Overall, the results indicate that generational differences were limited and activity-specific rather than widespread. When significant differences emerged, they primarily reflected greater acceptance among Generation X and Generation Y compared with Generation Z, suggesting that middle-aged generations may evaluate the practical usefulness of care robots more favorably than younger adults for selected instrumental daily activities.

### 4.3. Preferences for Type of Robot by Generation to Help in Daily Activities

**Figure 2:**
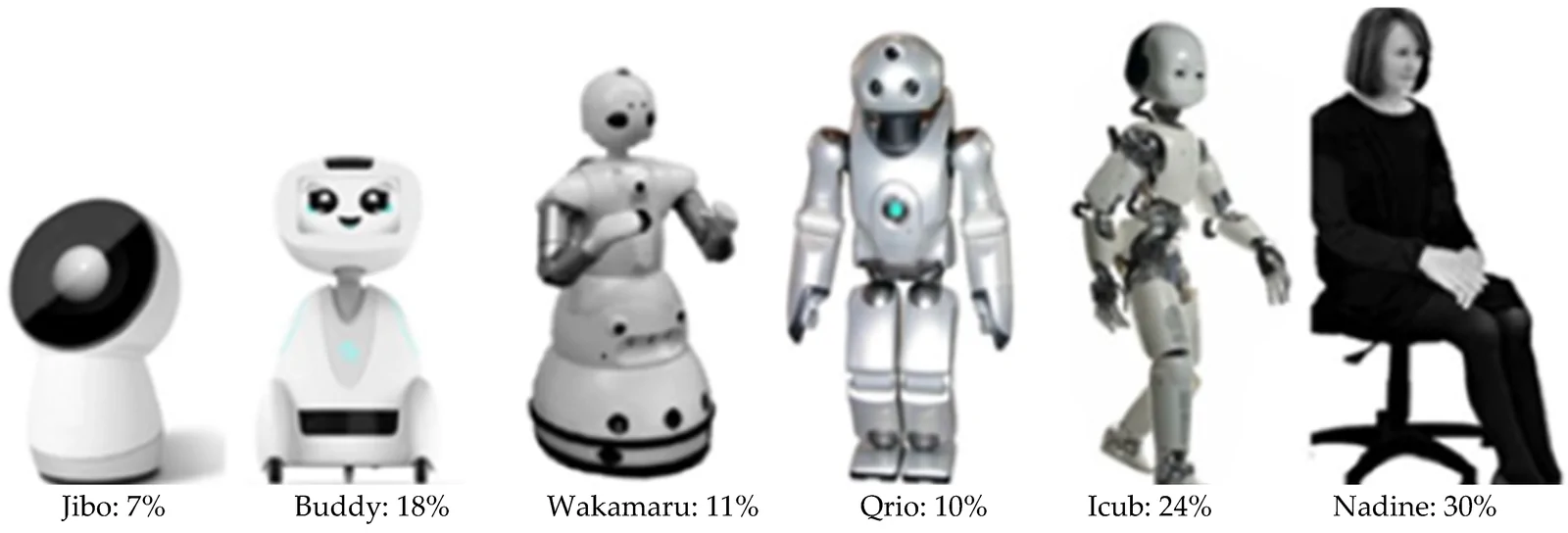
Types of Robots.

Firstly, the respondents chose the robot with more human similarities, as follows:

Preferences for the different robot designs (Figure 2) also varied across generational cohorts. Overall, Jibo received the lowest level of preference among all generations. In contrast, Nadine was the most preferred robot among Generation Z, Generation X, and Baby Boomers, indicating broad acceptance of a more anthropomorphic design. Buddy was also positively evaluated by Generation Z and Generation X, whereas Baby Boomers showed comparatively lower preference for this robot and higher preference for robots with a more human-like appearance. These results suggest that anthropomorphic characteristics may influence robot preferences differently across generations. A summary of the mean scores is presented in Table 3.

Hypothesis 2 (H2) examined whether generational cohort significantly influences perceptions of the usefulness of care robots for supporting Basic Activities of Daily Living (ADLs), as assessed using the Katz Index.

To test this hypothesis, mean differences among generational cohorts were analyzed using one-way ANOVA. Descriptive statistics and the corresponding mean comparisons for each ADL-related variable are summarized in Table 4.

### 4.4. Results for H2: Basic Activities of Daily Living (ADLs)

With regard to Basic Activities of Daily Living (ADLs), statistically significant differences were observed across generational cohorts for all activities assessed (*p* < 0.05). Nevertheless, the overall pattern of responses indicates that participants were generally reluctant to accept robotic assistance for intimate personal care activities, with mean scores reflecting disagreement or only limited acceptance across all generations.

Significant intergenerational differences were identified for the following activities:

- Bathing: Although all generations expressed negative attitudes towards robotic assistance with bathing, Generation Z reported significantly stronger disagreement than Generation X and Baby Boomers.
- Getting dressed: Generation X and Baby Boomers expressed significantly greater acceptance of robotic assistance with dressing than Generation Z, which generally disagreed with this application.
- Getting in and out of bed: Generation X demonstrated significantly higher acceptance of robotic assistance than Generation Z, which reported lower levels of agreement.
- Managing incontinence: Acceptance differed significantly across generations, with Generation X expressing greater willingness to receive robotic assistance than Generation Z.
- Assistance during meals: Generation X also reported significantly higher acceptance of robotic support during meals than Generation Z, whose responses remained predominantly negative.

Overall, the findings indicate that Generation X consistently demonstrated greater acceptance of robotic assistance for Basic Activities of Daily Living than Generation Z, particularly for activities involving physical support and personal care. Although statistically significant differences were observed across generations, acceptance of robotic assistance for intimate care tasks remained comparatively low among all groups, suggesting that bodily intimacy and personal vulnerability continue to represent important barriers to the adoption of care robots.

### 4.5. Preferences for Type of Robot by Generation to Help in Basic Needs Activities

First, the respondents chose the robot with more human similarities, as follows:

Robot appearance preferences for supporting Basic Activities of Daily Living (ADLs) also varied across generational cohorts. Consistent with the findings for Instrumental Activities of Daily Living (IADLs), Jibo received the lowest preference ratings across all generations. In contrast, Nadine was the most preferred robot among Generation Z, Generation X, and Baby Boomers, suggesting a generally favourable perception of more anthropomorphic robot designs. iCub was also positively evaluated by Generation Z and Generation Y. However, when considering assistance with Basic Activities of Daily Living, Generation X and Baby Boomers expressed comparatively stronger preferences for robots with a more human-like appearance than the younger generations. The descriptive statistics for robot appearance preferences across generations are presented in Table 5.

Almere Model:

Different Generations: Cross study.

The Almere Model is a technology acceptance framework designed specifically to predict how elderly users and residents in nursing homes accept assistive social agents (robots). Principal Components Analysis (statistical instrument) was used by transforming a large set of variables into a smaller one that nevertheless contains most of the information in the large set; 21 variables were transformed into three principal components. Component number 1 is Interaction with a Robot; Component number 2 is Usefulness of a Robot and Component number 3 is Anxiety and social influence. Comparison of mean differences in the variables of each component considering the variable generation was done.

Kaiser-Meyer-Olkin test was run; it is a statistical measure used in data science to determine if your dataset is suitable for factor analysis and verify the internal consistency of the components [40]. It obtained a KMO = 0.888 (KMO values between 0.8 and 1 indicate the sampling is adequate). Using the Varimax method to rotate the principal components allowed for an easier interpretation of the principal components, obtaining four principal components. The consistency was tested using Cronbach’s Alpha, regardless of the type of study, whether it is exploratory research, applied research, or scale development research; a criterion of 0.7 is universally employed [41]. After excluding variable Q17.14, Cronbach’s Alpha values were found to be higher than or equal to 0.7 (rounded) in all components. Table 6 presents a summary of the principal components, variables’ means, and Cronbach’s alpha (a statistical coefficient used to measure the internal consistency or reliability of a set of items of each principal component). Average Variance Extracted (AVE) and Composite Reliability (CR) were calculated and confirmed that AVE had values above 0.5 and CR values above 0.7 which indicate acceptable convergent validity [41]. AVE has the advantage of considering measurement error in variables [40], while CR provides a more unbiased reliability estimate than Cronbach’s alpha [42].

**Table 6:** Principal Components Analysis.

	Mean	Loading	%Variance Explained	Cronbach Alpha
PCA1-Interaction with a Robot				
Q17.4. I think using a robot is a good idea.	3.31	0.541		
Q17.5. A robot would make my life more interesting.	2.84	0.647		
Q17.6. It will be good to use a robot.	3.31	0.605		
Q17.9. I will enjoy hearing the robot talk to me.	3.85	0.613		
Q17.10. I will enjoy doing things with the robot.	2.74	0.699	16.8	0.870
Q17.11. I find using a robot fun.	3.01	0.655		
Q17.12. I find using a robot fascinating.	2.83	0.656		
Q17.15. I feel a robot will understand me.	2.64	0.712		
Q17.17. I think I would make a good impression if I used a robot.	2.61	0.722		
Q17.18. I would trust a robot’s advice.	2.55	0.667		
PCA2-Usefulness of a Robot				
Q17.7. I have everything I need to use a robot.	2.80	0.460		
Q17.8. I think a robot could adapt to my future needs.	3.32	0.676		
Q17.13 I find using a robot boring.	2.72	0.540		
Q17.16. I think a robot could help me with many things.	3.59	0.605	19.1	0.680
Q17.20. A robot could help in cases of loneliness and for those who live alone.	3.51	0.611		
Q17.21. It’s inevitable, in the future we will have robots to assist with basic and personal tasks.	3.73	0.725		
PCA3-Robot anxiety and social influence				
Q17.1. If I use a robot, I would be afraid of making mistakes while using it.	3.63	0.641		
Q17.2. I find robots scary.	3.15	0.858	13.1	0.773
Q17.3. I find robots intimidating.	3.08	0.851		
Q17.19. I think the people close to me wouldn’t approve of me using a robot.	2.97	0.646		

Likert scale (1—strongly disagree; 2—disagree; 3—not agree or disagree; 4—agree; 5—strongly agree). Mean comparison was analysed for all the variables in each principal component concerning “generation”, summary at Table 7.

**Table 7:** Principal Components and Mean Multiple Comparison-Post Hoc Tests Analysis.

Principal Components	Homogeneity of Variances		ANOVA		Variable Generation	Post Hoc Tests
					Z (18–29)	
					Y (46–61)	
	Levene	*p*-Values	F	*p*-Values	Baby Boomers (61+)	
PCA1-Interaction with a Robot						
Q17.4. I think using a robot is a good idea.	0.079	0.972	4.251	0.006	X: Mean: 3.56 Z: Mean: 3.11 (−0.453 *)	Scheffe
Q17.5. A robot would make my life more interesting.	0.272	0.845	1.128	0.339	(a)	N/A
Q17.6. It will be good to use a robot.	2 667	0.049	1.829	0.143	(a)	N/A
Q17.9. I will enjoy hearing the robot talk to me.	0.431	0.731	1.163	0.325	(a)	N/A
Q17.10. I will enjoy doing things with the robot.	2 103	0.101	3.111	0.028	X: Mean: 3.91 Z: Mean: 3.45 (−0.460 *)	Scheffe
Q17.11. I find using a robot fun.	0.365	0.778	0.633	0.595	(a)	N/A
Q17.12. I find using a robot fascinating.	1.507	0.214	0.188	0.904	(a)	N/A
Q17.15. I feel a robot will understand me.	1.655	0.178	0.138	0.937	(a)	N/A
Q17.17. I think I would make a good impression if I used a robot.	2.783	0.042	0.413	0.744	(a)	N/A
Q17.18. I would trust a robot’s advice.	2.390	0.070	0.502	0.681	(a)	N/A
PCA2-Usefulness of a Robot						
Q17.7. I have everything I need to use a robot.	0.707	0.549	2 254	0.083	(a)	N/A
Q17.8. I think a robot could adapt to my future needs.	2.433	0.066	1 878	0.135	(a)	N/A
Q17.13 I find using a robot boring.	0.751	0.523	0.683	0.564	(a)	N/A
Q17.16. I think a robot could help me with many things.	2.499	0.061	2.422	0.067	(a)	N/A
Q17.20. A robot could help in cases of loneliness and for those who live alone.	2.084	0.104	1.393	0.246	(a)	N/A
Q17.21. It’s inevitable, in the future we will have robots to assist with basic and personal tasks.	1.417	0.239	1.372	0.253	(a)	N/A
PCA3-Robot anxiety and social influence						
Q17.1. If I use a robot, I would be afraid of making mistakes while using it.	1.344	0.261	1.069	0.363	(a)	N/A
Q17.2. I find robots scary.	1.394	0.246	10.959	<0.001	X: Mean: 2.72 Z: Mean: 3.51 (0.788 *)	Scheffe
					Baby Boomers: Mean: 2.27 Z: Mean: 3.51 (1.236 *)	Scheffe
Q17.3. I find robots intimidating.	0.900	0.442	8.124	<0.001	X: Mean: 2.74 Z: Mean: 3.38 (0.633 *)	Scheffe
					Baby Boomers: Mean: 2.27 Z: Mean: 3.38 (1.104 *)	Scheffe
Q17.19. I think the people close to me wouldn’t approve of me using a robot.	1.511	0.213	3.372	0.020	X: Mean: 2.72 Z: Mean: 3.16 -(0.437 *)	Scheffe

* The mean difference is significant at the 0.05 level. (a) The mean difference is not significant at the level of 0.05. N/A: Not Applicable.

### 4.6. Generational Differences in Technology Acceptance of Care Robots (H3)

Hypothesis 3 (H3) examined whether generational cohort significantly influences the acceptance of care robots, including perceived usefulness and trust, as conceptualised by the Almere Model.

The analysis identified statistically significant differences across generations for several constructs related to anxiety, social influence, and behavioral intention, whereas no significant differences were observed for the perceived usefulness dimension.

The principal intergenerational differences were found for the following statements:

- “I find the robot scary.” Generation Z reported significantly higher levels of agreement than Generation X and Baby Boomers, both of whom generally disagreed with this statement.
- “I find the robot intimidating.” Generation Z also expressed significantly greater feelings of intimidation than Generation X, whose responses indicated general disagreement.
- “Using a robot is a good idea.” Although both Generation X and Generation Z agreed that using a care robot is a good idea, Generation X reported significantly higher levels of agreement.
- “I will enjoy using a robot.” Similarly, both generations expressed positive expectations regarding the future use of care robots; however, Generation X demonstrated significantly greater anticipated enjoyment than Generation Z.
- “People close to me would not approve of my using a robot.” Generation X generally disagreed with this statement, whereas Generation Z expressed significantly greater concern regarding potential disapproval from family members or other significant individuals.

Overall, the significant differences were concentrated within the Almere Model dimensions related to anxiety, social influence, and attitudes towards interacting with robots. In contrast, no statistically significant generational differences were observed for perceived usefulness. Across all generations, respondents generally agreed that care robots could be useful and helpful in supporting older adults and did not perceive them as inherently boring. Nevertheless, participants reported lower levels of confidence regarding their own readiness and capability to use such technologies.

Generation Z consistently reported the highest levels of anxiety towards care robots. Mean scores for the statements “I find the robot scary” and “I find the robot intimidating” were 3.51 and 3.38, respectively, indicating relatively neutral to moderately negative perceptions. By comparison, the corresponding mean scores were substantially lower among the older generations (Generation Y: 2.94 and 2.94; Generation X: 2.72 and 2.74; Baby Boomers: 2.29 and 2.29). These findings indicate that respondents aged 30 years and older generally perceived care robots as less frightening and less intimidating than participants belonging to Generation Z.

Taken together, these results provide partial support for H3. While perceptions of the usefulness of care robots were broadly similar across generations, significant differences emerged regarding the emotional acceptance of robotic technologies. In particular, younger participants reported higher levels of anxiety and stronger perceived social influence, whereas older generations appeared comparatively more comfortable with the prospect of interacting with care robots.

## 5. Discussion

The findings of this study provide key insights into how different generations in Portugal perceive the use of robots in elder care contexts, regarding activities of daily living, personal assistance, and human–robot interaction. Overall, the results indicate that attitudes toward robotic assistance are not uniformly positive or negative but instead vary according to the type of task involved, the perceived intimacy of care, and generational perspectives. These findings therefore reinforce the argument that acceptance of care robots cannot be analysed only through the lens of technological capability, but must also be understood through cultural, emotional, and social dimensions.

First, the results widely support the theoretical assumptions of Industry 5.0 and Society 5.0 discussed in the literature review. Both frameworks highlight human-centred technological integration, where robots and intelligent systems are expected to expand rather than replace human capabilities [8,12]. The findings suggest that Portuguese respondents generally recognize the potential usefulness of robots in practical and instrumental support tasks, particularly those linked to household management and assistance with instrumental activities of daily living (IADLs). Especially, respondents across generations showed higher acceptance for robots helping with cleaning the house, laundry, shopping, meal preparation, and transportation. These findings are consistent with Sawik et al. [22], who posited that aging societies increasingly require technological support systems capable of reducing caregiver burden and supporting independent living.

Second, nonetheless, the findings also demonstrate clear limits regarding acceptance. Whilst respondents were relatively open to robots supporting practical tasks, they expressed considerably lower acceptance for robots assisting with intimate and highly personal activities measured by the Katz Index, such as bathing, toileting, dressing, incontinence care, and feeding assistance. These findings powerfully align with the ethical concerns identified by WHO and Broadbent et al. [8,17], which argued that robotic elder care may become problematic when technologies begin replacing forms of care highly associated with empathy, dignity, and human presence. The results therefore reinforce the distinction between functional assistance and emotionally sensitive care tasks. In sum, Portuguese respondents appear more willing to accept robots as complementary support tools rather than substitutes for intimate human caregiving.

Third, an important contribution of this study is the identification of major intergenerational differences. Contrary to common assumptions that younger generations are automatically more technologically accepting, they would rather demonstrate more discomfort, fear and resistance toward care robots compared with Generation X and Baby Boomers [17]. In the Almere Model analysis, Generation Z perceived robots as significantly “scarier” and “intimidating” than older generations. Additionally, Generation Z was less supportive of robots assisting with intimate care activities and showed lower enthusiasm regarding interaction with robots.

These findings challenge simplified assumptions regarding digital natives and technology acceptance. Although younger generations are in general more familiar with digital technologies, such familiarity with smartphones and artificial intelligence applications does not necessarily translate into emotional acceptance of embodied robotic systems in caregiving contexts. This finding partially supports Heerink et al. [4], who claimed that technology acceptance is influenced not only by technical competence, but also by anxiety, perceptions of appropriateness, and emotional comfort.

Hence, respondents, particularly younger participants, may react negatively to robots that appear almost human but not fully human, generating feelings of discomfort or unease. Interestingly, however, participants simultaneously expressed a preference for robots with more human-like appearances, particularly Nadine and iCub. These findings illustrate the complex role of anthropomorphism in Human–Robot Interaction: while human-like appearance may enhance familiarity, perceived sociability, and emotional engagement, it may also raise expectations regarding robots’ social and emotional capabilities that are difficult to fulfil. Similar observations have been reported by Dautenhahn [39] and Bartneck et al. [37], who argued that anthropomorphic design can simultaneously facilitate emotional connection and influence users’ expectations, thereby affecting trust, comfort, and overall acceptance of robotic systems.

Fourth, the preference for more human-like robots was particularly strong among Generation X and Baby Boomers. These generations demonstrated higher acceptance of robotic assistance in both instrumental and basic care activities and preferred robots that visually resembled humans more closely. One possible explanation is that older generations may evaluate robots more pragmatically, mainly in the context of aging, declining autonomy, and future care needs. The literature review indicated that perceived usefulness is one of the strongest predictors of robot acceptance within the Almere Model [4,35]. Older respondents may therefore prioritize functionality and future support potential over concerns related to novelty or emotional discomfort.

Fifth, another important finding concerns the distinction between perceived usefulness and emotional trust. Although respondents generally agreed that robots could be useful, helpful, and increasingly important within future care systems, they remained considerably more cautious regarding emotional trust and intimate interaction. For example, respondents were relatively neutral or negative towards trusting a robot’s advice or believing that a robot could fully understand their emotional needs. These findings are consistent with the Almere Model, which conceptualises trust as a distinct determinant of technology acceptance rather than a simple consequence of perceived usefulness as posited by Heerink et al. [4]. They are further supported by Ham and Maeng [26], who demonstrated that acceptance of humanoid robots depends not only on perceptions of functional usefulness but also on users’ emotional trust and willingness to establish social relationships with robotic systems.

Sixth, the findings also reinforce the importance of cultural factors discussed throughout the literature review. Portugal, like many Southern European countries, maintains strong traditions of family-based caregiving and interpersonal support, where care is closely associated with emotional closeness, moral responsibility, and direct human contact. Consequently, resistance towards robotic assistance in intimate care activities may reflect not only technological concerns but also culturally embedded understandings of caregiving itself. Similar findings have been reported in other cultural contexts, where acceptance of care robots is strongly influenced by family-centred caregiving traditions, cultural values, and locally embedded social norms, as contended by Ahmad et al. [16] and Lima et al. [29]. These findings suggest that, despite differences between Asian and Southern European societies, cultural expectations regarding family responsibility and human-centred care may similarly shape attitudes towards robotic assistance.

Interestingly, despite expressing fear or discomfort toward robots, respondents did not totally reject their use. This ambivalence closely resembles the findings of Eurobarometer [3], which reported that Europeans generally recognize the benefits of robots whilst at once expressing concern regarding their role in sensitive care environments. The Portuguese sample appears to reflect this same duality: robots are accepted as useful assistants but not completely embraced as relational caregivers.

Seventh, the study also contributes methodologically by integrating the Almere Model with the Katz Index and the Lawton–Brody Scale. This approach enables technology acceptance to be analysed in relation to specific care activities and levels of functional dependence rather than solely through abstract attitudes towards technology. The findings indicate that acceptance varies considerably according to the type of activity involved. Respondents expressed greater acceptance of robots supporting Instrumental Activities of Daily Living associated with autonomy and household management, while remaining considerably more reluctant to accept robotic assistance for Basic Activities of Daily Living involving bodily intimacy and personal care. These findings are consistent with previous Human–Robot Interaction research demonstrating that older adults are generally more willing to accept robots for practical, household, and monitoring tasks than for intimate caregiving activities requiring empathy, dignity, and close interpersonal interaction as contended by Broadbent et al. [17], Sawik et al. [22] and Tobis et al. [34].

Finally, due to these insights, the findings additionally raise important implications for policymakers and developers within the context of Industry 5.0 and Society 5.0. Technological implementation in elder care cannot centre exclusively on efficiency or automation. Successful integration will require:

- Trust-building strategies;
- Human-centred robot design;
- Ethical safeguards;
- Emotional sensitivity;
- Culturally adapted implementation frameworks.

Robotic systems that are perceived as replacing human care may encounter considerable resistance. On the contrary, systems framed as complementary support tools designed to preserve autonomy and reduce caregiver burden may attain greater social acceptance. These social and policy-related issues are discussed below in detail. 

### 5.1. Sociocultural Implications of Generational Differences

One of the most significant contributions of the present study lies in challenging the widespread assumption that younger generations are naturally more willing to adopt emerging technologies simply because they have grown up in digitally mediated environments. Although Generation Z participants reported greater familiarity with digital technologies, smartphones and AI-based applications, they did not demonstrate the highest acceptance of care robots. In contrast, younger respondents frequently expressed greater fear, discomfort and emotional hesitation towards robotic assistance, particularly in intimate caregiving situations. These findings suggest that digital literacy and emotional acceptance represent distinct psychological constructs. Familiarity with digital technologies does not necessarily translate into willingness to establish relationships with embodied intelligent systems operating within emotionally sensitive contexts such as elder care.

From a sociocultural perspective, this distinction reflects broader transformations in the meaning of care within contemporary societies. Caregiving is not simply a technical activity but also a social and moral practice grounded in empathy, trust, reciprocity and interpersonal relationships. In Portugal, where family-based caregiving continues to play a central role, robots may therefore be perceived differently depending on the type of support they provide. Practical assistance with household management or medication may be viewed as compatible with traditional caregiving values, whereas intimate personal care remains strongly associated with human presence, compassion and emotional responsibility. These findings suggest that public acceptance of care robots is shaped not only by technological characteristics but also by culturally embedded understandings of dignity, family responsibility and appropriate caregiving. Consequently, future research should move beyond purely technological explanations and incorporate broader sociological and cultural perspectives when examining the acceptance of socially assistive robots.

### 5.2. Practical Implications for Robot Design, Healthcare Practice and Public Policy

The findings also generate important practical implications for developers, healthcare organizations and policymakers responsible for implementing robotic technologies within ageing societies. First, the clear distinction observed between acceptance of instrumental and basic care activities suggests that future care robots should prioritize functions that support independent living while preserving users’ autonomy and dignity. Developers should therefore adopt user-centered design approaches in which robots are conceived primarily as collaborative assistants rather than autonomous substitutes for human caregivers. Features related to household assistance, medication management, transportation support and communication may encounter fewer barriers to adoption than functions involving intimate physical care.

Second, healthcare organizations should recognize that successful implementation depends as much on organizational readiness and user education as on technological capability. Acceptance is likely to increase when older adults, family members and healthcare professionals are actively involved in the design, introduction and evaluation of robotic systems through participatory implementation strategies. Opportunities for direct interaction with robots, training programmes and transparent communication regarding their capabilities and limitations may reduce anxiety, improve trust and facilitate long-term adoption.

Finally, the findings have implications for public policy within rapidly ageing societies. Rather than promoting care robots as replacements for an overstretched healthcare workforce, policymakers should frame robotic technologies as complementary tools that strengthen existing models of person-centered care. Public investment should therefore support ethical governance frameworks, interoperability standards, professional training and equitable access to assistive technologies while ensuring that technological innovation remains aligned with the principles of autonomy, dignity, transparency and human oversight advocated by Industry 5.0 and Responsible Artificial Intelligence. Such an approach is likely to foster greater public confidence and facilitate the socially sustainable integration of care robots into future healthcare systems.

Overall, this study demonstrates that robotic elder care in Portugal is perceived neither as fully attractive nor entirely unacceptable. Acceptance is conditional, task-dependent, emotionally negotiated, and strongly influenced by generational and cultural factors. These findings reinforce the significance of maintaining a human-centred perspective when developing future care technologies and contribute to broader debates with respect to the role of robotics within aging societies.

## 6. Conclusions

This study aimed to analyse generational perspectives regarding the acceptance of robots in elder care within the Portuguese context using the conceptual frameworks of Industry 5.0 and Society 5.0. By integrating the Almere Model with the Katz Index and the Lawton and Brody Scale, the study explored not only broad attitudes toward care robots, but also how acceptance varies according to the type of assistance provided and the level of personal intimacy involved in care activities.

The findings demonstrate that Portuguese respondents generally recognize the potential usefulness of robots in supporting older adults, mainly in instrumental activities of daily living such as cleaning, laundry, shopping assistance, transportation, and meal preparation. These results propose that robotic technologies are increasingly perceived as valuable support tools capable of promoting autonomy, reducing caregiver burden, and supporting independent living in aging societies. This reinforces the human-centred vision suggested by Industry 5.0 and Society 5.0, where intelligent systems are intended to augment human well-being rather than replace human beings.

Nevertheless, the results also disclose important limits to acceptance. Respondents showed significantly lower acceptance of robots assisting with intimate and highly personal care activities such as bathing, toileting, dressing, feeding, and incontinence care. These findings confirm that elder care is not acknowledged only as a functional activity, but also as an emotional, relational, and culturally sensitive practice strongly associated with human presence, dignity, empathy, and trust.

One of the study’s most important findings concerns generational differences. Contrary to the assumption that younger generations are naturally more receptive to advanced technologies, Generation Z, in contrast, unveiled higher levels of discomfort, fear, and intimidation with regard to care robots compared with older generations. On the contrary, Generation X and Baby Boomers tended to show more pragmatic acceptance of robotic assistance, mainly when considering future aging-related support needs. These findings suggest that technology acceptance in elder care depends not only on digital familiarity, but also on emotional perceptions, cultural values, and perceived vulnerability.

The study also highlights the importance of trust in human–robot interaction. Although respondents recognized robots as potentially useful and inevitable within future care systems, emotional trust remained limited. Participants were cautious about fully trusting robots in sensitive situations or perceiving them as substitutes for meaningful human interaction. Therefore, acceptance appears conditional and context-dependent rather than unlimited.

Another relevant contribution concerns robot appearance and anthropomorphism. Respondents usually preferred robots with more human-like characteristics, particularly Nadine and iCub, particularly amongst older generations. Simultaneously, younger respondents frequently described robots as scary or intimidating. This indicates that the design and presentation of care robots may significantly influence social acceptance.

Overall, the findings suggest that robotic assistance in elder care is more likely to be accepted when positioned as complementary support rather than replacement for human caregivers. Technologies perceived as enhancing autonomy, facilitating daily activities, and reducing caregiver pressure may attain better social legitimacy than systems associated with emotional substitution or depersonalized care.

The study also contributes academically by extending research on robot acceptance into the Portuguese context, a Southern European country characterized by rapid demographic aging and strong family-based caregiving traditions. By linking technological acceptance with functional independence scales, the research offers a more idiosyncratic understanding of where and how robotic assistance may be socially acceptable.

In conclusion, the study demonstrates that robots are increasingly perceived as potentially valuable tools within elder care systems, but their acceptance remains conditional upon the nature of the care task, emotional trust, and cultural perceptions of caregiving. The future of robotic elder care in Portugal will likely depend not on replacing human relationships, but on developing technologies capable of supporting autonomy whilst preserving dignity, empathy, and meaningful human connection. Altogether, the findings suggest that successful implementation of care robots depends less on technological sophistication than on aligning robotic capabilities with users’ functional needs, emotional expectations and cultural values. Consequently, future care robotics should be designed as human-centred technologies that complement rather than replace interpersonal care.

## 7. Limitations and Future Research

This study has several limitations that should be considered when interpreting the findings. First, it employed a purposive sample drawn exclusively from the Lisbon Metropolitan Area, limiting the generalizability of the results to the wider Portuguese population. In addition, the unequal representation of generational cohorts, particularly the larger proportion of Generation Z respondents, may have influenced comparisons across groups. Second, the study relied on self-reported attitudes towards hypothetical care robot scenarios, and most participants had no direct experience interacting with socially assistive robots. Furthermore, the cross-sectional design precludes causal inferences regarding the relationship between generation and technology acceptance.

Future research should seek to replicate these findings using larger and more geographically diverse samples, with greater representation of older adults and individuals with caregiving experience. Longitudinal and experimental studies involving direct interaction with care robots would provide a better understanding of how trust, anxiety, and acceptance evolve over time. Cross-cultural comparisons, particularly between Southern European and Asian societies, together with qualitative approaches such as interviews or focus groups, could further explore the cultural, emotional, and ethical dimensions underlying the acceptance of care robots. Finally, more advanced analytical techniques, such as structural equation modelling, may help clarify the relationships between perceived usefulness, trust, functional support needs, and behavioral intention.

## Figures and Tables

**Table 1:** Sample characteristics.

*n* = 217		
Age	Frequency	Percentage
18–29 (Generation Z)	123	56.7
30–45 (Generation Y)	20	9.2
46–61 (Generation X)	48	22.1
+62 (Baby Boomers)	26	12
Gender		
Female	135	62.2
Male	81	37.3
Other	1	0.5
Completed Academic Qualifications		
Basic Education (up to 9th grade)	2	0.9
Secondary Education (up to 12th grade)	101	46.5
Bachelor’s Degree	82	37.8
Master’s/Doctorate	32	14.8
Family situation		
Single	140	64.5
Married	55	25.3
Divorced	20	9.2
Widower	2	1.0
With children	69	31.8
Without children	148	68.2
Work Contract		
Student or student and worker	112	51.6
Worker with fixed-term contract	36	16.6
Worker without fixed-term contract	26	12.0
Entrepreneur	43	19.8
Number of years of work		
Less than 6 months	53	24.4
6 to 11 months	14	6.5
1 to 3 years	37	17.1
4 to 10 years	26	12.0
More than 10 years	87	40.1
Net monthly income		
Until 500 euros	60	27.6
501 to 1000 euros	51	23.5
1001 to 2500 euros	57	26.3
2501 to 3500 euros	23	10.6
3501 to 5000 euros	16	7.4
More than 5000 euros	10	4.6

**Table 2:** Variables Lawton-Brody Mean Multiple Comparison and Post Hoc Tests.

						Post Hoc Tests	Types of Post Hoc Tests
Variables Lawton-Brody Scale: I Would Like a Robot:	Mean	Homogeneity of Variances		ANOVA		Variable Generation	
						Z (1829)	
						X (30–45)	
		Levene	*p*-Values	F	*p*-Values	Y (46–61)	
						Baby Boomers (61+)	
Q13.1. Use the phone for me	2.13	0.386	0.763	2.478	0.062	(a)	N/A
Q13.2. Help me use the phone	2.91	0.503	0.680	5.219	0.002	X: Mean: 3.40 Z: Mean: 2.63 (−0.775 *)	Scheffe
Q13.3. Do the shopping for me	3.21	0.225	0.879	1.440	0.232	(a)	N/A
Q13.4. Help me with the shopping	3.62	0.703	0.551	1.618	0.186	(a)	N/A
Q13.5. Prepare meals for me	3.46	0.794	0.499	0.727	0.537	(a)	N/A
Q13.6. Clean the house for me	4.19	4.327	0.006	2.938	0.034	X: Mean: 4.44 Z: Mean: 4.03 (−0.419 *)	Scheffe/Dunett C.
						Y: Mean: 4.60 Z: Mean: 4.03 (−0.575 *)	Scheffe/Dunett C.
Q13.7. Take care of the laundry for me	3.99	2.626	0.052	3.480	0.017	Y: Mean: 4.65 Baby boomers: Mean: 3.75 (−0.900 *)	Scheffe
						Y: Mean: 4.65 Z: Mean: 3.86 (−0.792 *)	Scheffe
Q13.8. Provide transportation for me	3.30	2.171	0.093	2.357	0.073	(a)	N/A
Q13.9. Take care of my medication	2.92	0.429	0.732	1.962	0.121	(a)	N/A
Q13.10. Keep me company	2.53	0.008	0.999	0.146	0.932	(a)	N/A
Q13.11. Manage my finances	2.84	0.428	0.733	0.233	0.874	(a)	N/A

* *p*-value < 0.05 =>The mean difference is significant at the 0.05 level. (a) *p*-value > 0.05 => the mean difference is not significant at the level of 0.05. Likert scale (1—strongly disagree; 2—disagree; 3—not agree or disagree; 4—agree; 5—strongly agree). N/A: Not Applicable.

**Table 3:** Robot preference by generation to help in daily activities.

	Z (18–29)	Y (30–45)	X (46–61)	Baby Boomers (61+)
Nadine	25%	23%	45%	45%
Icub	23%	27%	28%	29%
Buddy	24%	27%	10%	3%
Qrio	14%	8%	3%	3%
Wakamaru	8%	12%	9%	10%
Jibo	7%	4%	5%	10%

**Table 4:** Variables Katz Scale: Mean Multiple Comparison and Post Hoc Tests.

						Post Hoc Tests	Types of Post Hoc Tests
Variables Katz Scale: I Would Like the Help of a Robot for:	Mean	Homogeneity of Variances		ANOVA		Variable Generation	
						Z (18–29)	
						Y (30–45)	
		Levene	*p*-Values	F	*p*-Values	X (46–61)	
						Baby Boomers (61+)	
Q15.1. Bathing	2.27	1.506	0.214	8.701	<0.001	X: Mean: 2.77 Z: Mean: 1.92 (−0.857 *)	Scheffe
						Baby Boomers: Mean: 2.93 Z: Mean: 1.92 (−1.017 *)	
Q15.2. Getting dressed	2.52	0.841	0.473	7.641	<0.001	X: Mean: 3.18 Z: Mean: 2.17 (−1.012 *)	Scheffe
						Baby Boom: Mean: 3.00 Z: Mean: 2.17 (−0.831 *)	Bonferroni
Q15.3. Going to the bathroom	2.47	1.047	0.373	9.357	<0.001	X: Mean: 3.16 Z: Mean: 2.09 (−1.066 *)	Scheffe
						Baby Boom: Mean: 3.00 Z: Mean: 2.09 (−0.907 *)	Scheffe
Q15.4. Getting in and out of bed	2.68	1.752	0.158	5.801	<0.001	X: Mean: 3.32 Z: Mean: 2.37 (−0.945 *)	Scheffe
Q15.5. Dealing with incontinence	2.76	0.493	0.687	5.369	<0.001	X: Mean: 3.32 Z: Mean: 2.48 (−0.835 *)	Scheffe
Q15.6. During meals	2.81	2.928	0.035	4.756	0.003	X: Mean: 3.41 Z: Mean: 2.53 (−0.875 *)	Scheffe/Dunett C.

* *p*-value < 0.05 => The mean difference is significant at the 0.05 level. Likert scale (1—strongly disagree, 2—disagree, 3—not agree or disagree, 4—agree, 5—strongly agree).

**Table 5:** Robot preference by generation to help in basic needs activities.

	Z (18–29)	Y (30–45)	X (46–61)	Baby Boomers (61+)
Nadine	27%	25%	48%	52%
Icub	27%	32%	24%	24%
Buddy	15%	14%	10%	3%
Qrio	13%	7%	5%	3%
Wakamaru	11%	18%	6%	10%
Jibo	6%	4%	8%	7%

## Data Availability

The data presented in this study are not publicly available because they contain information that could potentially compromise participant privacy and confidentiality. The dataset includes individual responses collected from human participants and is therefore subject to ethical and privacy restrictions. The anonymized dataset supporting the findings of this study is available from the corresponding author upon reasonable request.
